# Supplementary material for: Pulp obtained after isolation of starch from red and purple potatoes (Solanum tuberosum L.) as an innovative ingredient in the production of gluten-free bread
Source: PLoS One. 2020 Sep 18;15(9):e0229841. doi: 10.1371/journal.pone.0229841 (PMC7500602; doi:10.1371/journal.pone.0229841)
Supplement: Attachment — Submitted filename: PONE-D-19-31585_reviewer.doc [file pone.0229841.s001.doc]

Journal: **PLOS ONE**

The authors of the manuscript raise a very important, from the point of view of the circular economy, aspect of by-product management that arises after the isolation of starch from red and purple potatoes. Among other things, due to the fact that the remaining pulp is rich in health-related compounds, both in the nature of polyphenols and dietary fiber, a possible direction of its use could be its use as an innovative fraction of dietary fiber, for example in the production of gluten-free bread. The obtained results allow us to plant a successfully applied test of pulp application, whereas red potato pulp is characterized by better health potential.

**In conclusion, the manuscript is interesting, zarówno ze wzgledu na aspekt poznawczy, jak I aplikacyjny. I recommend the manuscript to be published. I suggest minor editorial changes:**

Line 3 - put the star symbol "*" next to Gumul - this person is the author of correspondence

Line 37 – it is suggested to add at least a literature reference in which the pulp production method would be described.

Line 120 – 4000 rpm – spin speed should be expressed as the value of centrifugal force "g"

Line 121 – reference should be given

Line 126,128,129,130,132 – the bibliography entry should be standardized

Line 143, 195 – Marconi et al. should be Matrconi *et al.*

W Tab. 1, 2, 3, 4 - g/100g d.m. should be g 100g^-1^ d.m.

significance levels (letters) should be placed in superscript

- there should be a dots after the titles of the tables and figures

- in table 4 – microgramy/kg should be µg kg^-1^

- in table 4 the acrylamide values should be given to the first decimal place

- Line 351 - at the end of a sentence put a dot.
