# Supplementary material for: Pulp obtained after isolation of starch from red and purple potatoes (Solanum tuberosum L.) as an innovative ingredient in the production of gluten-free bread
Source: PLoS One. 2020 Sep 18;15(9):e0229841. doi: 10.1371/journal.pone.0229841 (PMC7500602; doi:10.1371/journal.pone.0229841)
Supplement: Attachment — Submitted filename: Response to rewievers.docx [file pone.0229841.s002.docx]

Additional Editor Comments:

In the study was performed sensory analysis, but that no information is provided

regarding participant consent (it is necessary for prospective studies, even in

cases where the study has been deemed on &ldquo;low risk&rdquo; by an ethics

committee). Please complete this information.

**According to the regulations in Poland, the consent of the bioethics committee is required only with respect to medical experiments (Regulation of the Minister of Health and Social Welfare on detailed rules for appointing and financing and the mode of operation of bioethics committees - Dz. U. 1999 No 47 item 480). In turn, the Act on the professions of a doctor and a dentist (Journal of Laws 2019, item 537 as amended - hereinafter: JoL) distinguishes two types of medical experiments - therapeutic and research. According to article 21 section 2 JoL "A medical experiment is the introduction by a doctor of new or only partially tested diagnostic, therapeutic or prophylactic methods ...". And "A research experiment is primarily aimed at extending medical knowledge ...". (Article 21(3) JoL). In addition, in accordance with the rules of the Pharmaceutical Law Act (Journal of Laws 2019, item 499, as amended), the tasks of the Bioethics Committee also include providing opinions on clinical trials of medicinal products (Article 37l, paragraph 1).**

**Gluten-free breads containing potato pulp are not a medicinal product but a food product. It follows from the above, that in the case of the studies presented in our article the consent of the bioethics committee was not required. Sensory evaluation is widely used in our studies on food products and the committee's consent was never required when publishing the results of such studies. Moreover, it does not appear in scientific publications of other authors known to us.**

**All the panelists who participated in sensory evaluation voluntarily agreed to be involved in the survey and were informed about the type of additive included in bread formulation.**

**Example papers;**

***Jarosław Korus, Mariusz Witczak, Rafał Ziobro, Lesław Juszczak, Hemp (Cannabis sativa subsp sativa) flour and protein preparation as natural nutrients and structure forming agents in starch based gluten-free bread Lwt-Food Science and Technology, 2017, DOI: 10.1016/j.lwt.2017.05.046***

***Magdalena Krystyjan, Dorota Gumul, Rafał Ziobro, Marek Sikora, The Effect of Inulin as a Fat Replacement on Dough and Biscuit Properties, Journal of Food Quality, 2015, 38 (15), 305-315***

***Gumul D, Areczuk A., Ziobro R., Ivanisova E, Zieba T. The influence of freeze-dried red and purple potatoes on content of bioactive polyphenolic compounds and quality properties of extruded maize snacks. Quality Assurance and Safety of Crops and Foods. 2017, doi. 10.3920.QAS. 2016.1055.***

Reviewer’s comments

Review Comments to the Author

**Thank you very much for the comments. We have tried to correct the manuscript according to the suggestions. Please find below the answers for specific comments:**

Reviewer #1:

General: I suggest replacing ‘gluten free’ with ‘gluten-free’;. The authors use both forms at work. This should be unified.

**applied**

Lines 23-24: This is not entirely true. The use of whole-grain flour, e.g. from

amaranth or quinoa, also provides bioactive compounds. Besides, are only

antioxidants important compounds? Please, in the summary and in the introduction,

approach the subject more broadly.

**Appropriate text was added to the abstract and the introduction.**

Keywords: Please avoid repeating the words used in the title.

**Corrected according to the suggestion.**

Lines 39-41: How much potato pulp is produced during starch production? Please

mention the second by-product, i.e. potato juice and (briefly) the possibilities of

its use.

**Appropriate text was added to the introduction.**

General: The authors point out in the introduction that the gluten-free diet is low

in minerals, protein, etc. Then they add potato pulp to enrich the bread with fiber,

which hinders the absorption of nutrients. Can the authors explain how increasing

dietary fiber content can help in qualitative malnutrition?

**The content of minerals and other nutrients was increased by the applied addition of potato pulp. Appropriate text explaining this issue was added to the introduction.**

Lines 105; 163; 169 etc.: ‘freeze dried’ -> ‘freeze-dried’

**corrected**

Line 106:

total starch or maybe just one of them? Potato pulp contains much more water than

starch. Did the replacement include water content?

**corrected**

Line 120: Please convert rpm to g or rcf

**corrected**

Section ‘Acrylamide content’ The authors correctly described the

results obtained, however, please refer to food safety. What are the limits for AA?

**Appropriate text was added to the section.**

Reviewer #2

Line 3 - put the star symbol ‘*’ next to Gumul - this person is the author

of correspondence

**corrected**

Line 37 – it is suggested to add at least a literature reference in which the

pulp production method would be described.

**Additional reference was added.**

Line 120 – 4000 rpm – spin speed should be expressed as the value of

centrifugal force “g”

**corrected**

Line 121 – reference should be given

**Additional reference was added.**

Line 126,128,129,130,132 – the bibliography entry should be standardized

**corrected**

Line 143, 195 – Marconi et al. should be Matrconi et al.

**corrected**

Tab. 1, 2, 3, 4 - g/100g d.m. should be g 100g-1 d.m.

**corrected**

significance levels (letters) should be placed in superscript

- there should be a dots after the

**corrected**

titles of the tables and figures

- in table 4 – microgramy/kg should be µg kg^-1^

**corrected**

- in table 4 the acrylamide values should be given to the first decimal place

**corrected**

- Line 351 - at the end of a sentence put a dot.

**corrected**
